# From cooperative to uncorrelated clogging in cross-flow microfluidic membranes

**DOI:** 10.1038/s41598-018-24088-6

**Published:** 2018-04-09

**Authors:** R. van Zwieten, T. van de Laar, J. Sprakel, K. Schroën

**Affiliations:** 1 Physical Chemistry and Soft Matter, Wageningen University, Wageningen, The Netherlands; 2 Laboratory of Food Process Engineering, Wageningen University, Wageningen, The Netherlands

## Abstract

The operational lifetime of filtration membranes is reduced by the clogging of pores and subsequent build-up of a fouling or cake layer. Designing membrane operations in which clogging is delayed or even mitigated completely, requires in-depth insight into its origins. Due to the complexity of the clogging process, simplified model membranes fabricated in microfluidic chips have emerged as a powerful tool to study how clogs emerge and deteriorate membrane efficiency. However, to date, these have focussed solely on dead-end filtration, while cross-flow filtration is of greater practical relevance at the industrial scale. As such, the microscopic mechanisms of clogging in crossflow geometries have remained relatively ill-explored. Here we use a microfluidic filtration model to probe the kinetics and mechanisms of clogging in crossflow. Our study exposes two findings: (i) the primary clogging rate of individual pores depends only on the trans-membrane flux, whose strong effects are explained quantitatively by extending existing models with a term for flux-controlled flow-enhanced barrier crossing, (ii) cross-membrane flow affects the pore-pore communication, leading to a transition from correlated to uncorrelated clogging of the membrane, which we explain qualitatively by deriving a dimensionless number which captures two essential regimes of clogging at the microscale.

## Introduction

Membrane filtration is a crucial unit operation in a very wide variety of industrial processes, ranging from milk separation^1^, pharmaceutical fractionation^2^, waste-water treatment^3^ and the removal of biological contaminants from donor blood^4^. Despite its ubiquitous use, one of the major causes of membrane performance deterioration, i.e. clogging and the subsequent build-up of filter cakes or fouling layers, remains a major challenge in the successful design of membrane operations. Clogging leads to large losses in productivity, due to reductions in flux and the cleaning required to keep the membrane functioning^5^. Different strategies exist to keep membranes operational, such as back pulsing, back flow reversal, various cleaning cycles and ultimately complete replacement^6,7^. And while these procedures can enhance the operational efficiency to some extent, neither address the underlying mechanism of clogging^8^.

Traditionally, three mechanisms for clogging are identified^9,10^; (i) *sieving*, in which a single particle whose dimensions exceed the pore size is trapped at the pore entrance and immediately causes it to become blocked, (ii) arching, in which multiple particles form a configurational arch over a pore, a scenario found in the passage of dense particulate flows through constrictions and (iii) flow-induced aggregation, where multiple particles form an agglomerate by means of irreversible aggregation, either within the flow or at a pore wall, which builds gradually over time until it completely clogs a pore. In particular in the latter scenario, clogging is a complex phenomenon in which a large variety of factors play a role. Such as particle-particle and particle-wall interactions, colloidal stability, hydrodynamic interactions, the confinement ratio, particle concentration, membrane geometry, etc.^5,11–13^.

To disentangle this multidimensional phase space with the aim to arrive at a more predictive and generic description of aggregation-induced clogging^14,15^, microfluidic micromodels in which excellent and independent control can be obtained on these parameters, have proven valuable tools to study clogging at the microscale^16–18^. For example highlighting how particle interactions^5,17,19^, polydispersity^20^ and contaminants^9^ play a role, the governing role of particle flux^16^, the effects of complex collective dynamics^21,22^, how pore geometry influences clogging rates^18^ and the effects of particle softness^23–25^.

Interestingly, while most industrial processes use a crossflow filtration strategy^26,27^, in which a flux is established both across and over the membrane, these microfluidic model experiments have primarily focussed on dead-end filtration geometries^5,9,14–25,28^. In industry, cross-flow geometries are used as they are found to remain operational longer, but the question remains what the exact mechanisms of this performance enhancement at the microscopic scale are. Optimizing the performance of crossflow systems is typically done by studying the decay of the crossmembrane flux as a function of time^29–31^. While valuable, these measurements do not provide insight into what occurs in and on the membrane that leads to its failure.

In this paper we study clogging in a microfluidic clogging device that mimics cross-flow filtration. We study the effect of cross-flow velocity and trans-membrane flux on clogging by quantitative microscopic imaging of our cross-flow microfluidic device. We find that the rate of clogging only depends on the trans-membrane flux, which we explain quantitatively by extending existing models with a transition-state term that introduces the hydrodynamic enhancement of particle-wall and particle-particle sticking. Moreover, we show that the cross-flow across the membrane mainly influences the cake build-up that follows after the formation of a clog. A higher cross-flow flux reduces the rate of cake build-up and thus delays the communication and clogging between neighboring pores mediated by the filter cake. This results in a transition from cooperative to uncorrelated membrane clogging; we qualitatively treat these effects by introducing a new dimensionless number describing this transition.

## Results and Discussion

**Figure 1:**
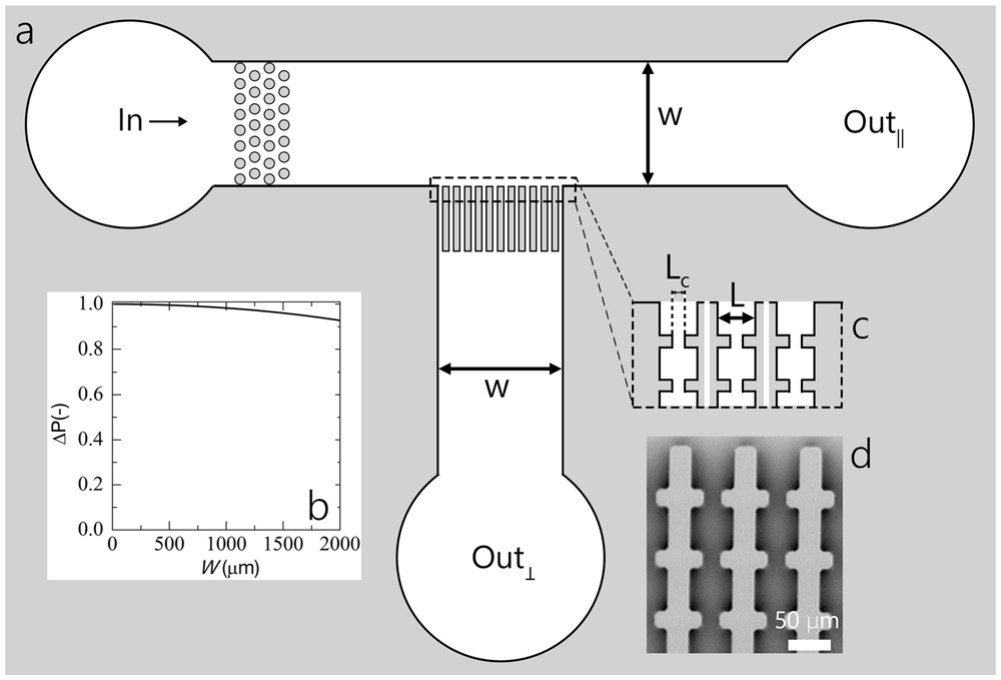
Overview of the multiplexed cross-flow filtration micromodel: (**a**) the device geometry, (**b**) the relative pressure drop over the total width of the multi-pore membrane, (**c**) detailed zoom-in to illustrate the membrane pores in more detail. Each pore features 19 constrictions in series, each of identical geometry, along the flow direction. The membrane consists of 30 pores within the same device; this allows 30-repeat measurements in a single experiment and the exploration of pore-pore communication. (**d**) Scanning Electron Microscopy (SEM) picture of the membrane micromodel.

To study clogging in cross-flow filtration we design a cross-flow microfluidic filtration device, based on previous dead-end microfiltration designs^9,17,18,23^. An overview of the device is given in Fig. 1. A membrane is placed at the centre of a t-shaped cross-flow channel (with *W* = 2.1 mm) and consists of 30 parallel pores (width of pore, *L* = 50 *μ*m), each having 19 constrictions in series, with *L*_*c*_ = 20 *μ*m. All constrictions within the 30 parallel pores are equal and have the same 90 degree entrance angle (as shown in Fig. 1c). The distance between the constrictions in a single pore along the trans-membrane flow direction is kept constant at 50 *μ*m. As we aim to study clogging by aggregation, we need to eliminate large contaminants^9^ from our fluid streams by means of a coarse pre-filtration element. This contaminant filter is composed of a hexagonal micropillar array a few hundred micrometers before the membrane (circles in Fig. 1a). As a reference measurement, we also study dead-end filtration in the same device, by leaving the cross-flow outlet (*out*_||_ in Fig. 1a) closed, so that all flow is directed through the membrane.

We flow a dilute suspension (3% wt) of polystyrene particles (radius *a* ≈ 1.2 *μ*m, such that the confinement ratio is roughly a factor 8) through our micromodel using a pressure controlled set-up, where we can vary the pressure to change the cross-flow and trans-membrane fluxes (see Materials and Methods for details). Initially all pores transport dispersion, until a clog forms at one of the constrictions within the pore. Following the initial clog formation, the clog acts as a sieve such that fluid passes but particles do not; as a result a filtration cake forms, first inside the pore and eventually spilling over the top of the membrane structure. As in our previous study on dead-end filtration, we accelerate the clogging process to within a realistic experimental time window by inducing a weak depletion attraction between our particles by the addition of silica nanoparticles as depletants (see Materials and Methods section for more detail).

Inherently, the cross-flow design implies a pressure drop across the membrane from the first to the last pore. We have designed our devices in such a way to minimize this pressure drop such that each pore experiences almost the same initial conditions. We calculate the pressure drop across the 30-pore membrane using the Hagen-Poiseuille law^32^:

1 $${P}={{P}}_{0}-\frac{32\eta \bar{u}}{{{d}}^{2}}({x}-{{x}}_{0})$$

where *P*_0_ is the pressure at the first pore, *η* is the viscosity of the fluid, $\bar{u}$ is the average particle velocity, *d* the hydraulic diameter of the tube, *x*_0_ the start of the membrane and *x* the position along the membrane surface ranging from *x*_0_ to the end of the membrane. We approximate *d*^2^ by the area of the entire membrane. As shown in Fig. 1b, the relative pressure drop is small for all conditions studied here, where *W* represents the width of the entire membrane surface.

Our experiments are run at fixed pressure as this ensures that clogging of one or more pores does not change the trans-pore flux for the remaining open pores. A pressure controlled experiment allows the total flux to decrease as the flow resistance increases due to clog formation, which ensures that the flux per open channel remains constant. We directly measure the trans and cross-membrane fluxes at all applied pressures by collecting the outcoming fluid on a analytical balance when a particle-free fluid is flowed through the device. We average these data by collecting data for at least 90 minutes to ensure a high statistical certainty on these numbers. In this way, we determine directly both the cross-flow flux (*Q*_||_) and the trans-membrane flux (*Q*_*T*_).

### Primary clogging rate

In order to obtain statistical information about the rate at which our cross-flow micromodel clogs we determine the time at which each separate pore clogs. To detect clogging events in individual pores we make use of the change in transmitted light intensity in a pore at the singular event of clogging. Initially, the pore is permeated with the suspension, which creates a medium grey level in our brightfield tranmission imaging set-up due to weak scattering of the particle-laden fluid. As a pore clogs we observe a distinct change: downstream from the clog the intensity increases as only fluid permeates the clog and thus no scattering centres (particles) are found downstream, while the intensity decreases upstream as a filter cake builds. We threshold this raw data and subsequently use automated image analysis to detect when and where a clogging event occurs. We repeat each experiment in triplicate to ensure reasonable statistical certainty on the data discussed below.

**Figure 2:**
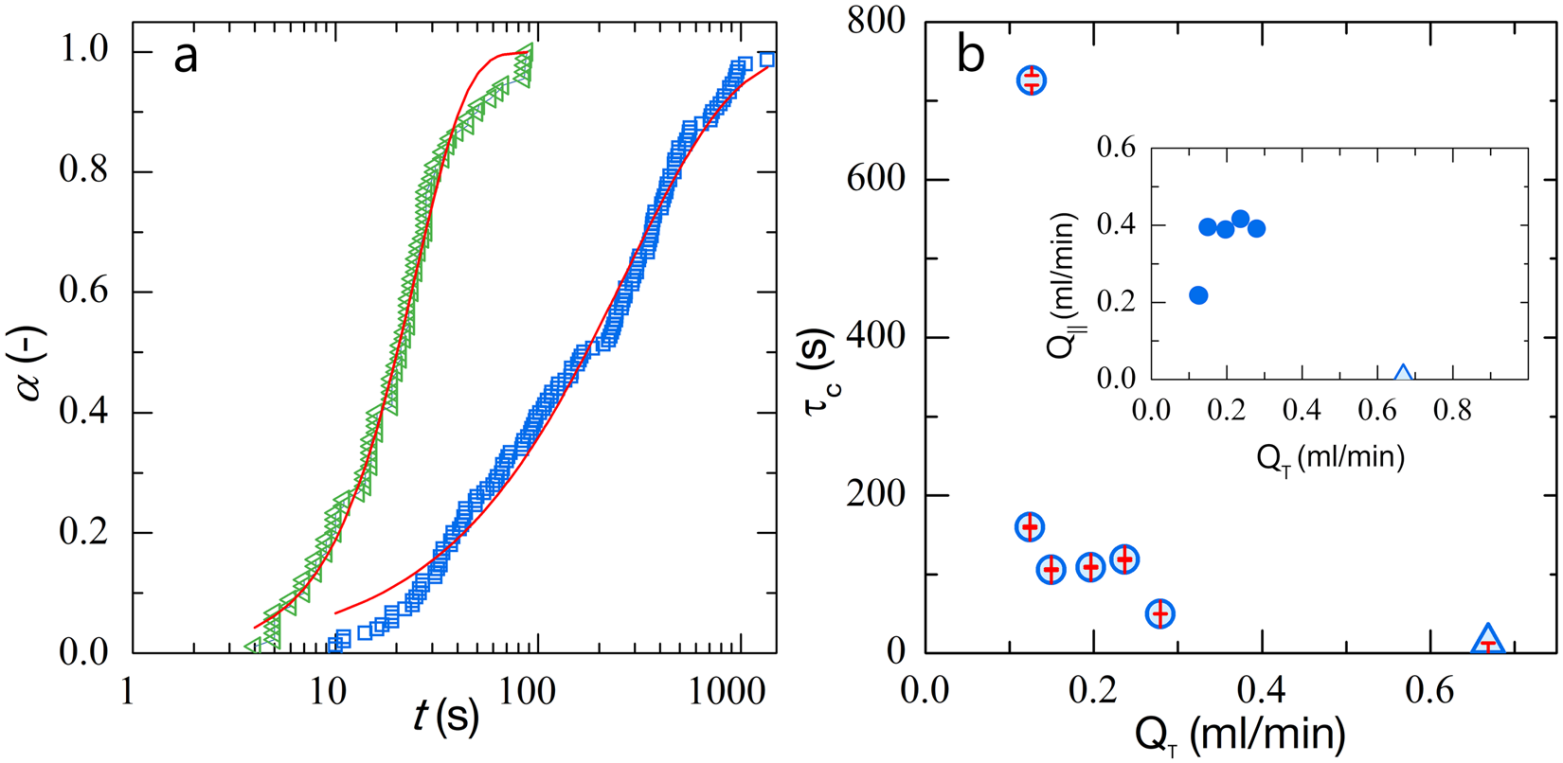
Fraction of clogged channels *α* as a function of time at a flow rate of 0.13 and 0.28 ml/min. (**a**). (**b**) Characteristic clogging time *τ*_*c*_ as a function of trans-membrane flux, red bars show the 95% confidence intervals for each data point. The light blue triangle in (**b**) represents the clogging time for dead-end filtration. The inset in (**b**) shows the relation between cross and trans membrane fluxes for all experiments described in this paper.

We start by calculating the fraction of channels that have clogged, *α*, as a function of time after the suspension first enters the membrane. We observe that as time progresses more and more pores become clogged, with *α* showing a gradual increase over time (see Fig. 2a, blue squares). As we increase the trans-membrane flux, *Q*_*T*_, we see that *α* grows more rapidly as a function of time, i.e. clogging occurs faster (see Fig. 2a, green triangles, where *Q*_*T*_ is two times that of *Q*_*T*_ for the blue squares).

To describe the dependence of *α* as a function of time we use the Weibull model^33^, which describes clogging as a cascade of individual failure processes and is commonly used to describe a wide variety of failure kinetics which obey weakest-link scaling, including those found in dead-end filtration^18^. The Weibull distribution is formulated as:

2 $$\alpha \,=\,1-\exp {[\frac{t}{{\tau }_{c}}]}^{\beta }$$

where *τ*_*c*_ is a characteristic clogging time and *β* is the stretch exponent, which indicates the statistical distribution of failure times for individual pores. We fit the model to our data using least-squares regression, and find good quantitative agreement at all applied flow rates (red lines in Fig. 2a). We see that for all flow rates the value of *β* is close to unity, which indicates clogging occurs through a Poisson process with a well-defined characteristic failure time. Also here we note, that we have observed similar behavior in a pure dead-end filtration geometry^18^.

We measure *τ*_*c*_ for a given flux and plot these as a function of the trans-membrane flux, which was determined independently. We see that as the trans-membrane flux (*Q*_*T*_) increases *τ*_*c*_ decreases dramatically (Fig. 2b). We observe that *τ*_*c*_ strongly depends on *Q*_*T*_, but how does it depend on the cross-flow flux? As these experiments are all performed at constant pressure to account for flow rate differences due to clogging it is impossible to independently vary the cross-flow and trans-membrane flow, as shown in the insert in Fig. 2b. However, as the expectation is that cross-flow increases the time till clogging, and we observe the opposite correlation, we postulate that in these cases the dominating factor governing the failure time is the trans-membrane flow. This is supported by the fact that for the two cases with the lowest values of *Q*_||_, we find almost 3 orders of magnitude difference in *τ*_*c*_, where the blue triangle in Fig. 2b represents a reference experiment in the same micromodel operated in dead-end mode, without any cross-flow over the membrane. We can also compare our results to those previously found in our dedicated dead-end microfiltration model^18^. At comparable flow rates and conditions we find that *τ*_*c*_ is in the same order of magnitude for both the cross-flow system and our dead-end system^18^. While a direct comparison is difficult due to different particle sizes and attraction strengths, the closest comparison shows that *τ*_*c*_ ≈ 150 s for cross-flow and *τ*_*c*_ ≈ 160 s for dead-end filtration^18^. Which again seems to indicate that *τ*_*c*_ here depends on *Q*_*T*_, and not on the cross-flow flux *Q*_||_.

**Figure 3:**
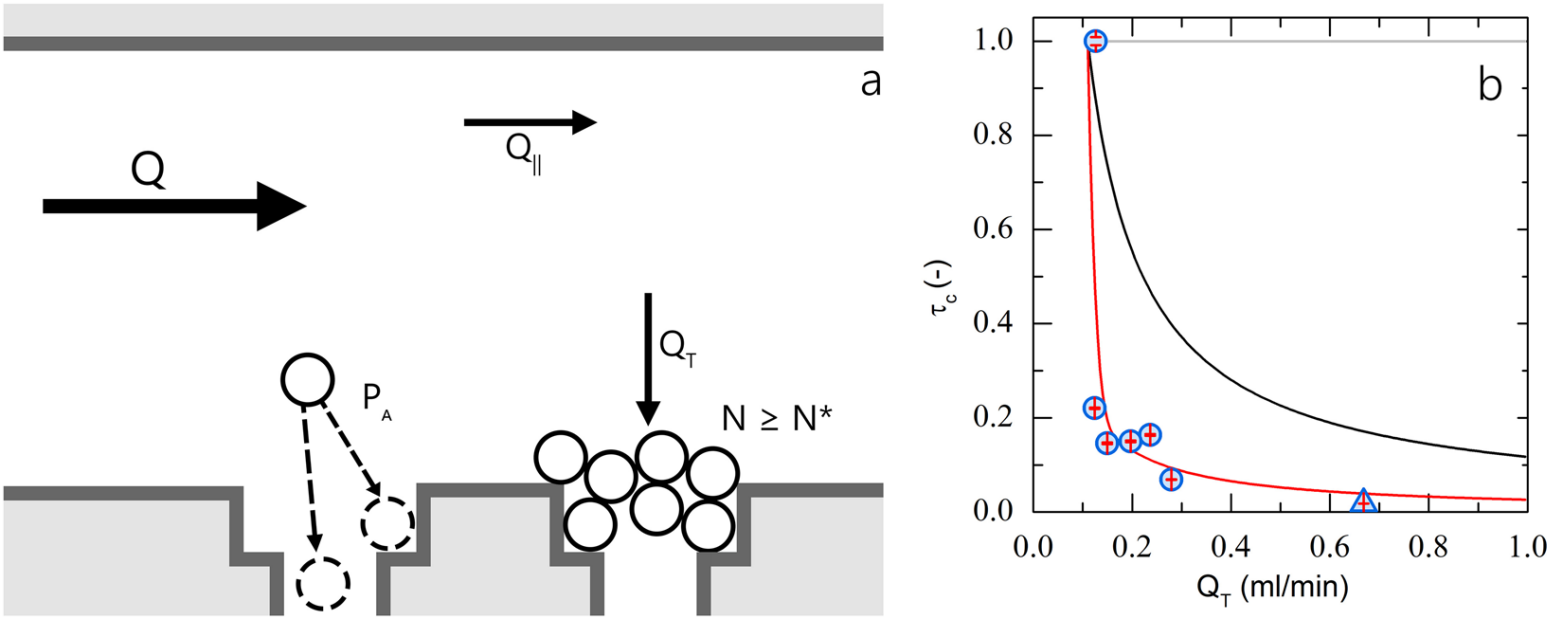
(**a**) Schematic representation of the clogging process, see main text for details. Normalised clogging time as a function of trans-membrane flux (**b**), where the symbols are experimental data with red bars indicating the 95% confidence intervals, the red line is a fit to the model developed in this article, the black line indicates the prediction from previous theory in which hydrodynamic-enhancement of *P*_*a*_ is not accounted^17^ and the grey horizontal line indicates the dependency found when taking the model of^17^ and introducing the effects of residence time without hydrodynamic-enhancement of aggregation.

To explain the steep decay in clogging time *τ*_*c*_ on the transmembrane flux *Q*_*T*_ we first consider the explanation for clogging postulated previously by others^17^. Here it is proposed that clogging occurs when a critical number of particles have passed the constriction, which would predict a dependency of *τ*_*c*_ ∝ 1/*Q*_*T*_; as shown by the black line in Fig. 3b, the experimentally observed decay is much steeper. Clearly, some additional effects must be taken into account. The previous explanation did not consider that the longer a particle resides in a pore, the higher its probability of sticking to a wall becomes. If these residence time effects are taken into account, with the residence time *t*_*R*_ scaling with 1/*Q*_*T*_, a flux-independent clogging rate would be expected (grey horizontal line in Fig. 3b). It is clear that some additional effect must be considered to explain the experimentally observed steep decay of the clogging time with trans-membrane flux.

We reformulate the model proposed previously^17^ to take the hydrodynamic enhancement of particle aggregation into account using a transition-state argument. We consider clogging as the consequence of sequential particle sticking events to the pore walls or particle agglomerates previously formed on those walls. When a critical number of particles N^*^ has accumulated on the walls, a clog will form. Note that we ignore the finite, but small, probability that a particle detaches from a growing agglomerate, i.e. we only consider particle association occurring at a rate *k*_*a*_ and the rate of dissociation *k*_*d*_ ≈ 0 is assumed to be negligible. This assumption will be discussed in more detail below in the context of cake formation and erosion. We presume that particle sticking is a thermally-activated and mechanically-enhanced process, governed by thermal fluctuations and thus probabilistic in nature.

We identify two opposing effects. First, the probability of particle aggregation depends on its residence time in or near the constriction; the longer a particle resides near the constriction, the larger the probability it will stick and become immobilized. Thus, this predicts a decrease in the particle sticking probability *P*_*a*_ with trans-membrane flux *Q*_*T*_ (see Fig. 3a for a schematic illustration). Secondly, at higher fluxes the flow velocities and thus viscous drag forces exerted on the particles will be larger. These viscous forces can push particles against the wall, thereby enhancing the rate at which they cross the activation barrier for aggregation, and thus increasing their sticking probability with trans-membrane flux. The first effect predicts an increase in clogging time with *Q*_*T*_, while the second decreases the clogging time.

For thermally-activated particle association onto a wall or existing pre-clog, the association process exhibits a Poisson distribution. The probability that a particle remains free *P*_*f*_ decays with the time it has been in proximity to the wall or pre-clog as:

3 $${{P}}_{{f}}={e}^{-{k}_{a}t}$$

where *k*_*a*_ is the association rate constant. The probability that a particle is stuck after a residence time *t*_*R*_ thus becomes:

4 $${P}_{a}=1-{e}^{-{k}_{a}{t}_{R}}$$

Where the residence time *t*_*R*_ depends on the mean local flow velocity $\bar{v}$ and constriction length *l* as

5 $${t}_{R}=\frac{l}{\bar{v}}$$

We can estimate the mean flow velocity from the flux as $\bar{v}={Q}_{T}/An$, where *A* is the cross-sectional area of one constriction and *n* = 30 the number of pores in parallel in our membrane micromodel design. Substituting the result above, yields for the sticking probability:

6 $${P}_{a}=1-\exp [\frac{-{k}_{a}lAn}{{Q}_{T}}]$$

The rate of particle aggregation events *k*_*a*_ was established previously to be significantly enhanced by viscous forces acting on the particles in the shear flow^18^. These viscous forces can be approximated as the Stokes force on a spherical particle $F=\bar{v}\zeta =\frac{{Q}_{T}\zeta }{An}$, with *ζ* = 6*πηa* the Stokes drag coefficient in which *η* is the fluid viscosity and *a* the particle radius. Within the transition-state picture^34,35^ of thermally-activated and mechanically-enhanced processes the association rate constant can be defined as

7 $${k}_{a}={k}_{a,0}\,\exp [\frac{F\delta }{{k}_{B}T}]={k}_{a,0}\,\exp [\frac{{Q}_{T}\zeta \delta }{{k}_{B}TAn}]$$

in which *k*_*B*_*T* is the thermal energy, *δ* the activation length, corresponding to the range of the attractive Van der Waals forces responsible for aggregation, typically in the order of one *nm*, and *k*_*a*,0_ the aggregation rate constant in the absence of shear forces, which is set by the energy barrier that keeps the particles stable in suspension.

Finally, we suppose that clogging requires the sequential aggregation of a critical number of particles N^*^, as shown in the right part of Fig. 3a. The number of stuck particles *N* increases with time as:

8 $$N={Q}_{T}\rho {P}_{a}t$$

in which *ρ* is the number density of particles in the fluid stream that crosses the membrane at flux *Q*_*T*_, which stick with a probability *P*_*a*_(*t*_*R*_). At the clogging time *t* = *τ*_*c*_, the critical number of particles is reached *N* = N^*^, such that the clogging time can be approximated as

9 $${\tau }_{c}=\frac{{N}^{\ast }}{{Q}_{T}\rho {P}_{a}}$$

Using Eqs 6, 7 and 9 we can now explore the dependence of the characteristic clogging time on the trans-membrane flux, accounting for both the reducing effects of shorter residence times at higher fluxes and the enhancing effects of larger viscous forces at higher *Q*_*T*_.

As can be seen from Fig. 3b, our proposed theory provides a good qualitative explanation of the observed data. Thus indeed, an accurate predictive description of pore clogging must take the force-enhancement of barrier crossing into account. We note that to describe our data, we need a value of *δ* which is significantly smaller than that expected based on the geometry of our system (*δ* = 2^−11^ m), this difference could lie in the simplification made in the calculation of *P*_*a*_ using Eq. 6. Namely, this probability does not take into account that only a small fraction of all the particles that flow through the membrane gets in close enough contact with the membrane walls to be able to stick. Thus, while this approach is approximate and mean-field in nature, it appears to capture qualitatively the underlying physics. This also calls for the development of new and more precise models to describe these effects from a microscopic picture of the entire process; this is however out-of-scope for this paper. Based on these values we find a sharp increase in *P*_*a*_ as the transmembrane flux increases, going from almost zero at quiescent conditions (*P*_*a*_ ≈ 0.001) to unity at higher fluxes (*P*_*a*_ = 1 for *Q*_*T*_ 0.2 ml/min); indeed, we find that at rest the dispersion is colloidally stable which must imply a very low value of *P*_*a*_ in the absence of hydrodynamic forces. We see that the behavior of *P*_*a*_ perfectly mirrors that of *τ*_*c*_, where at low flow rates sticking is highly unlikely (resulting in a long clogging time), and as the flow rate increases *P*_*a*_ ≈ 1 and *τ*_*c*_ decreases rapidly. Thus, the hydrodynamic enhancement of particle aggregation appears to be the dominant term in governing the rate of clogging.

Interestingly, our experimental data of clogging time versus flux cannot be described by considering only the passage of a critical number of particles, but requires taking hydrodynamically-enhanced barrier hopping into account, in contrast to the previously published data of Wyss *et al*.^17^ that shows a simple reciprocal dependency on the flux. These two results are thus in apparent contradiction. Based on the work of Dressaire *et al*.^9^, it has become clear that (i) commercial dispersions often used in these experiments contain a very small (ppm) fraction of large contaminants which (ii) lead to clogging by sieving which dominates the clogging behavior if no precautions are taken to remove them prior to the dispersion arriving at the microfluidic model. Indeed, if large impurities clog a pore singularly and this determines the clogging rate, a simple reciprocal dependence on flux must be found. In both of these previous works, carboxylated polystyrene particles are used, prepared by emulsion polymerisation, in which it is known that small amounts (1 in 1 million)^9^ of much larger particles are inevitably present. In this case, clogging occurs through a completely different mechanism. This could thus be the reason of the apparent contradiction remarked upon above. By contrast to these previous studies, we have introduced a micropillar filter at the entrance of our device, with the specific goal of removing such large impurities, thus enabling us to probe clogging by aggregation of the primary small particles under study. This hypothesis is further substantiated by the large difference in clogging times, despite working at similar volume fractions and pore-particle size ratios; e.g. in the work of Dressaire and Wyss, typical clogging times are in the range of 0.1–100 seconds^9,17^, while in our case for repulsive suspensions clogging takes one or two orders-of-magnitude longer, with clogging times ranging from half an hour to several hours^18^.

### Pore communication

Based on our experimental results, the transition-state model and comparison to previous results for dead-end filtration with similar particle dispersions^18^, it becomes clear that application of a cross-flow flux does not impact the primary clogging process in these experiments. In our case, primary clogging, which is the singular event in which the flow of particles through the pores becomes blocked, occurs within the membrane, where the eroding effects of the cross-flow are negligible. However, while cross-flow does not alter the primary clogging rate of individual pores, it does effect the rate of failure of the membrane as a whole.

To date, the clogging of membrane micromodels has been described as the uncorrelated clogging of *n* individual pores. However, the growth of the filter cake on top of the membrane as the result of a single pore clogging, may affect the clogging rate of its neighbors. In such a scenario, clogging may become strongly cooperative in which the clogging of a single pore enhances, through the filter cake, the clogging of neighboring pores and thus the failure of the membrane as a whole.

**Figure 4:**
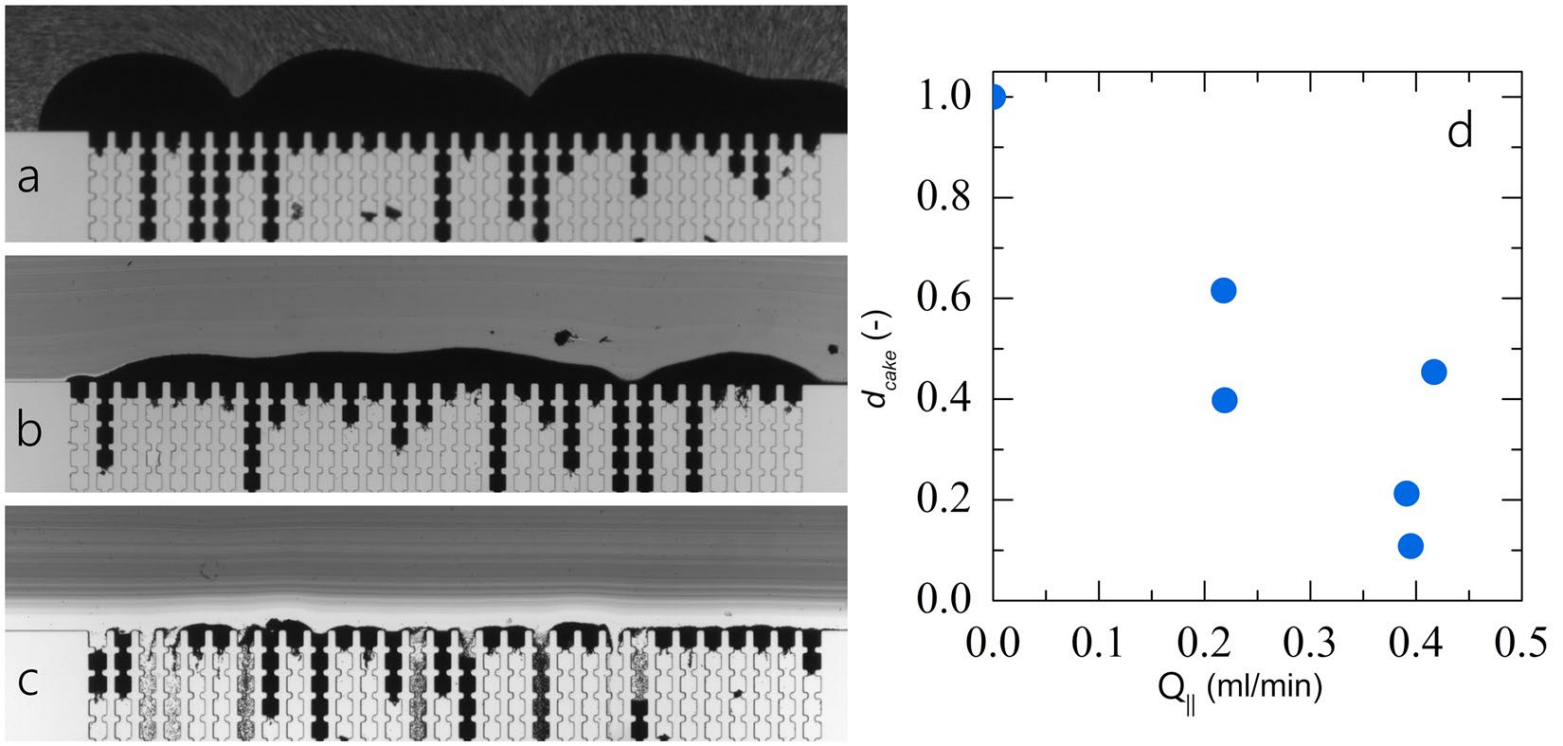
Representative images illustrating the formation of a filtercake during (**a**) dead-end filtration in our cross-flow micromodel, (**b**) cross-flow filtration at a cross-flow rate of 0.219 ml/min and (**c**) 0.395 ml/min. (**d**) Cake thickness *d*_*cake*_ as a function of cross-flow flux.

We first investigate how the cross-flow induced shear forces acting on a growing filter cake effect its thickness. We visually notice a significant reduction in the cake layer build-up when the cross-flow velocity is increased (see Fig. 4a–c). From these microscopy images we can calculate the average cake thickness over the entire membrane structure; indeed, we find a strong decrease in the cake layer thickness as the cross-flow flux increases (Fig. 4d).

The differences in cake build-up between dead-end (Fig. 4a) and cross-flow filtration (see Fig. 4b,c) are significant in two ways. First, the delay in cake growth reduced the growth in hydrodynamic resistance across the membrane and thus delays the time at which the pressure-flux relation deteriorates to such an extent that cleaning of the membrane is required in an industrial setting^26,27^. Secondly, if the filter cake mediates the communication between pores and thus the cooperativity of the failure of the membrane, cross-flow could change the mode of membrane failure.

To explore the extent of pore-pore communication we ask the question how the clogging of a given pore affects the clogging rate of its neighbors. To answer this question, we determine the distance, counted in number of pores, between to consecutive clogging events. If the clogging is uncorrelated, the distance between two clogging events should show a flat distribution, where the probability of finding the next clogging event some distance Δ*x* away shows no preference for neighboring clogs. By contrast, if the clogging is correlated and cooperative, the probability of finding the next clogging event should be largest for the shortest distance Δ*x* and decay rapidly after that.

For a fair evaluation of the distance-dependence of the next-clogging probability, we must account for the relative occurrence of possible pore distances by calculating a weighted probability function, where we assign weights to each value for Δ*x*, based on how often this possibility can occur in our micromodel. This is essentially a weighting for the configurational entropy of the clogging process. For example, while almost every pore (except the most left and most right one) will have two pores at Δ*x* = 1, only 2 out of the 30 pores will have a possible Δ*x* = 29.

**Figure 5:**
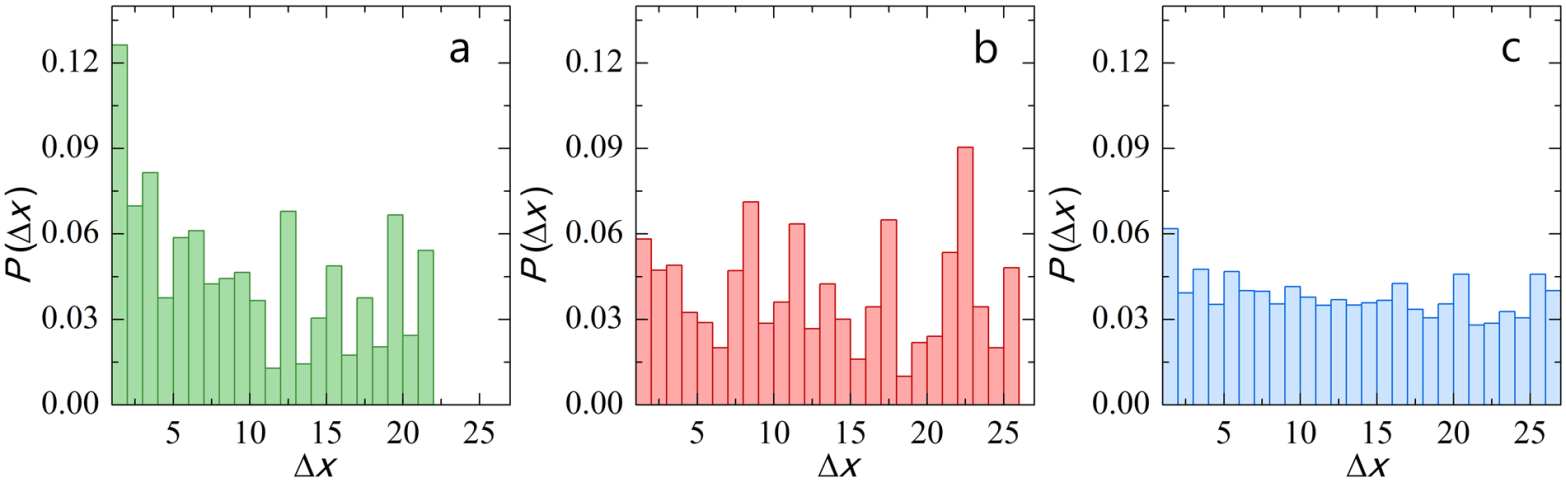
Weighted probability distributions of the distance to the next clogging event, where the distance Δ*x* is expressed as the number of pores between two consecutive clogging events, for (**a**) dead-end filtration for purely repulsive particles, data as in^18^ (**b**) dead-end filtration for a suspension with attractive forces (*U* ≈ 4 *k*_*B*_*T*, data as in^18^ and (**c**) cross-flow filtration for all combined data in this paper. The probability distributed are weighted for the conformational entropy as dictated by the device design, as described in the text.

We indeed find that for dead-end filtration of a purely repulsive system (Fig. 5a, data adapted and reproduced from)^18^ clogging is strongly correlated, with the probability peaking at Δ*x* = 1, mediated by overhanging filter cake^18^. Interesting, we find that two changes in the set-up both reduce the correlations and lead to uncorrelated membrane failure: (i) increasing the primary clogging rate by making the particles weakly attractive (Fig. 5b, data adapted and reproduced from)^18^ and introducing strong cross-flow (Fig. 5c).

To explain this transition from cooperative to uncorrelated clogging of multipore membranes we must realize that two different rates play a role. On the one hand we have the primary clogging rate which dictates the rate at which particle-aggregation induced clog formation leads to pore blockage. On the other hand we have the rate of filter cake build-up; if the rate of cake build-up and spill-over into neighboring pores is much faster that the primary clogging rate, the clogging process becomes cooperative. By contrast, if primary clogging occurs more rapidly than cake-induced clogging, we must find an uncorrelated membrane failure processes.

The typical time it takes a single pore to clog is prescribed by Eq. 9 as: *τ*_*c*_ = N^*^/(*Q*_*T*_*ρP*_*a*_). The characteristic time *τ*_*fc*_ it takes a growing filter cake to reach the neighboring pore, in a simplified picture, is a balance between growth rate ${\dot{h}}_{+}$ (in m/s) and the cross-flow induced erosion rate ${\dot{h}}_{-}$ (in m/s), as ${\tau }_{fc}=l/({\dot{h}}_{+}-{\dot{h}}_{-})$. In this relation, *l* is the average depth within the pore at which a clog occurs, which itself is governed by a Poisson distribution for a device with many identical constrictions in series. The rate of cake growth depends on the flux through the pore *Q*_*T*_/*n*, the volume fraction of particles in the incoming stream *ϕ* and the maximum close-packing volume fraction *ϕ*_*c*_ as:

10 $${\dot{h}}_{+}=\frac{{Q}_{T}\varphi }{n{A}_{p}{\varphi }_{c}}=\frac{{Q}_{T}\rho {a}^{3}}{n{A}_{p}{\varphi }_{c}}$$

in which *A*_*p*_ is the cross-sectional area of the pore. The rate of cake erosion ${\dot{h}}_{-}$ will depend on the height of the cake on top of the membrane, the shear forces acting on the particles within the cake governed by *Q*_‖_ and details of the particle-particle interactions in the cake, following a similar transition-state argument as derived above. For the purposes here of arriving at a qualitative understanding of the transition from cooperative to uncorrelated clogging, we simply assume that the erosion rate is a positive function of the cross-flow velocity ${\dot{h}}_{-}({Q}_{\parallel })$; a more quantitative analysis of the erosion rate is too involved for the current purposes and may be topic for future study.

This allows us to define a dimensionless clogging number *Cl* as the ratio of the primary clogging time and the cake build-up time as:

11 $$Cl=\frac{{\tau }_{c}}{{\tau }_{fc}}$$

which defines the transition from correlated and cooperative membrane failure when *Cl* ≫ 1 to uncorrelated failure of the membrane by a independent sequence of primary clogging events when *Cl* ≪ 1.

With the above we can define the clogging number for crossflow filtration as:

12 $$Cl=\frac{{N}^{\ast }}{{Q}_{T}\rho {P}_{a}l}(\frac{{Q}_{T}\rho {a}^{3}}{{\varphi }_{c}{A}_{p}}-{\dot{h}}_{-}({Q}_{\parallel }))$$

which can be simplified for dead-end filtration when ${\dot{h}}_{-}({Q}_{\parallel })=0$ to:

13 $$Cl=\frac{{N}^{\ast }{a}^{3}}{{P}_{a}l{\varphi }_{c}{A}_{p}}$$

which interestingly depends only on the pore geometry (N^*^, *l* and *A*_*p*_) and the particle stability (*P*_*a*_).

Indeed, our experiments highlight how either increasing the sticking probability *P*_*a*_, by introducing attractions (Fig. 5b), or increasing the erosion rate ${\dot{h}}_{-}({Q}_{\parallel })$, by increasing ${Q}_{\parallel }$ (Fig. 5c), can lower *Cl* to such an extent that the cooperativity of the membrane failure disappears, leading to an increase in the membrane lifetime.

## Conclusion

In this paper we explored the effects of trans- and cross-membrane fluxes on the clogging of pores and failure of multi-pore membranes. We find that the primary clogging occurs within the pores where it is unaffected by the cross-flow. Moreover, we show how the trans-membrane flux has a much steeper adverse effect on the clogging rate than predicted previously. We quantitatively explain these effects by introducing a term to account for the hydrodynamic enhancement of particle aggregation into existing clogging theories. Finally, we show how the crossflow does not alter the primary clogging rate but alters the communication between pores in a multi-pore membrane. We find that the extent of pore-pore communication can be tuned by changing the ratio of the primary clogging rate versus the rate of cake build-up, leading to a transition from cooperative to uncorrelated clogging. We rationalize these finding by deriving a new dimensionless clogging number and support this concept using experimental observation of the pore-pore correlations. This exploratory study on the microscopic mechanisms of cross-flow filtration are performed in highly idealised conditions. Exploring the effects of non-uniform pore sizes, pore-pore fluid connections and the effects of particle properties and dispersity would be an interesting avenue for further research, as this would bridge the gap between the model approach taken here and the real world of membrane filtrations in industrial applications. Nonetheless, our findings give rise to deeper insight into the dominant mechanisms that govern clogging and membrane failure, and could form a stepping stone for the more efficient design of membrane operations with enhanced operational lifetime.

## Materials and Methods

### Experimental system

We use a 3 wt% suspension of monodisperse polystyrene particles, prepared by dispersion polymerization^36^, with a diameter of 2.4 *μm* in a density matching mixture of 63 vol% water and 37 vol% deuterium oxide. We add 0.1 wt% pluronic F127 as a surfactant to sterically stabilize the particles and to prevent particle absorption to the PDMS walls^5^. The particle synthesis proceeds as follows: in 150 ml butanol we dissolve 17 ml styrene monomer, 2.34 g poly(vinylpyrrolidinone)-k30, 0.64 g dioctyl sulfosuccinate sodium salt (AOT) and 0.170 g 2,2-azobis(2-methylpropionitrile) (AIBN). We mix and purge the solution with nitrogen for 20 minutes to remove all oxygen and leave the flask under vacuum to perform the reaction. The reaction is left to proceed overnight at 70 °C. The particles are cleaned by repeated centrifugation and resuspension cycles in which we slowly exchange from the alcoholic reaction solvent to the aqueous solvent mixture in our experiments.

We induce weak attractive forces between the polystyrene particles, as this reduces the total time required for clogging and thus increases the efficiency of the experimental procedure, while maintaining the overall clogging behavior; these effects for the identical system have been explained in detail previously^18^. We introduce these attractive forces using depletion interaction, via the addition of silica nanoparticles (Ludox TM-40)^37^, and estimate the resulting attraction strength as^38^:

14 $$\frac{U}{{k}_{B}T}=\frac{3}{2}\frac{a}{{a}_{s}}{\varphi }_{s}$$

where *U* is the attraction strength in units of the thermal energy *k*_*B*_*T*, *a* and *a*_*s*_ are the radii of the larger polystyrene particles and smaller Ludox particles respectively (*a*_*s*_ ~ 7 *nm*) and *ϕ*_*s*_ is the volume fraction of the Ludox particles. For our experiments we choose an attraction strength *U* = 2 *k*_*B*_*T*, in between those used previously^18^. Prior to each experiment, the particle suspension is sonicated to make sure no particle aggregation occurs at rest.

### Microfluidic experiments

We fabricate the microfluidic devices using standard soft litography methods^39^. The pattern is replica-templated in Sylgard 184 silicone rubber at a mixing ratio of base to catalyst of 10:1. We cure devices at 65 ° for at least 1.5 hours, and subsequently bond them onto glass slides using a oxygen-plasma treatment. In all of our experiments, the devices have a height of 40 *μ*m. We use pressures of 28, 45, 63, 98, 133 and 168 mbar, controlled with an accuracy of 0.1 mbar, applied with an Elveflow OB1-MK3 pressure controller, resulting in transmembrane fluxes of 0.13, 0.23, 0.12, 0.15, 0.20 and 0.28 ml/min, respectively. The experiments are run at constant pressure such that the flux per pore remains constant, irrespective of the clogging of its neighbors^40^. One reference experiment was performed in dead-end geometry with the same device, at the highest inlet pressure of 168 mbar, while keeping the outlet orthogonal to the membrane closed. This results in a transmembrane flux of 0.67 ml/min.

We calculate the upper bound of the fluid Reynolds number to be *Re*_*f*_ ≤ 3, thus ensuring laminar flow conditions. The particle Reynolds number is computed to be *Re*_*p*_ ≤ 10^−3^ ensuring no inertial effects that may contribute to complexity in the clogging phenomena. The particle Peclet number is *Pe*~10^4^ indicating a flow dominated by advection. The microfluidic experiments are imaged using standard brightfield transmission microscopy (Zeiss Axiovert 200) equipped with a ThorLabs USB camera, at an acquisition rate of 1 Hz. The raw image time sequences are analyzed using a set of custom image processing routines as described previously^18^.
